# Exploring the effectiveness of podcasts in improving sexual health among young people: Findings from a qualitative study

**DOI:** 10.1371/journal.pone.0343514

**Published:** 2026-03-27

**Authors:** Yixuan Zou, Albie Sharpe, Maddison Stratten, Daniel Demant

**Affiliations:** 1 School of Public Health, Faculty of Health, University of Technology Sydney, Sydney, New South Wales, Australia; 2 HIV & Related Programs Unit (HARP), Illawarra Shoalhaven Local Health District, Wollongong, New South Wales, Australia; 3 School of Public Health and Social Work, Faculty of Health, Queensland University of Technology, Brisbane, Queensland, Australia; University of Colombo Faculty of Medicine, SRI LANKA

## Abstract

**Background:**

Sexual health promotion among young people remains an urgent global priority. Despite robust public health systems, young people living in Australia continue to experience unmet needs in sexual health promotion due to cultural stigma, lack of relatable content, and insufficient inclusivity for marginalised populations. Podcasts are gaining attention as a flexible, relatable medium that may address these gaps, yet little research has explored their effectiveness in sexual health promotion from young people’s perspectives. This study aimed to explore how the On The Couch podcast supports young people’s sexual health.

**Methods:**

This qualitative study conducted five focus groups with 18 participants aged 18–24 residing in Australia who had listened to the On The Couch sexual health podcast. Participants were recruited via university channels, social media, and podcast platforms, and reflected diverse cultural and educational backgrounds. Data collection involved semi-structured focus groups conducted both online and face-to-face. Transcripts were analysed using Braun and Clarke’s thematic analysis approach to identify themes and sub-themes. Ethical approval was granted by the Greater Western Human Research Ethics Committee (Reference: 2023/ETH02655) with ratification from the University of Technology Sydney Medical Research Ethics Committee.

**Results:**

Three major themes emerged from the analysis: (1) Podcasts significantly improved knowledge and shifted attitudes toward sexual health through relatable storytelling and emotional resonance. However, translating awareness into sustained behavioural change remained challenging, largely due to cultural and structural barriers. (2) Young people valued podcasts for their conversational tone, accessibility, and convenience, but highlighted the need for multimodal enhancements such as visual aids and transcripts. (3) Participants offered specific recommendations for improving podcast design, advocating for increased youth co-creation, authentic narratives, interactive formats, and culturally sensitive content.

**Conclusions:**

The study demonstrates that podcasts hold considerable promise for youth-focused sexual health promotion by providing relatable, emotionally engaging content. However, their effectiveness in changing behaviour is contingent upon addressing cultural barriers. Future podcasts should be developed with young people as co-creators, ensuring content aligns closely with their lived experiences, learning preferences, and social realities. This approach may enhance the role of podcasts as an effective medium in digital sexual health promotion.

## Introduction

### Background

Sexual health is a fundamental aspect of young people’s overall wellbeing and development; it remains an under-addressed public health priority worldwide [1]. Good sexual health is broader than just being free from sexually transmissible infections (STIs). Instead, it is defined as a satisfying, positive, and respectful sexual experience free from violence and exploitation [2]. This definition highlights how an individual’s sexual health is a function of complex and interconnecting biological, psychological, and social factors in their life [3]. However, young people often experience elevated vulnerability to sexual and reproductive health risks due to a combination of limited formal education, self-gender identity, evolving social norms, and inconsistent access to healthcare [3]. According to the US Centres for Disease Control and Prevention (CDC), young people aged 15–24 years account for nearly half of all new sexually transmissible infections (STIs) globally each year [4]. While sexual health information is increasingly presented digitally and its reach is expanded, young people continue to face significant barriers in locating reliable, culturally relevant sexual health resources [5]. Misinformation, difficulty evaluating sources, and stigma around seeking sexual health help persist across contexts. These barriers are further intensified for marginalised groups, including LGBTQIA+ youths and those from culturally diverse backgrounds, which means they often experience exclusion from mainstream health services and limited access to inclusive knowledge [6].

In Australia, despite relatively robust public health infrastructure, sexual health among young people remains a public health concern (Australian Government Department of Health and Aged [7]). Data from the Kirby Institute [8] indicates that youth aged 15–29 continue to have the highest STIs rates nationally, particularly for chlamydia, which accounts for around 70% of all cases and corresponds to 60,563 notifications. Additionally, many young people report limited understanding of key sexual health topics such as contraception use, consent, pleasure, and communication in relationships, which reflects gaps in comprehensive sexual health education and ongoing stigma around accessing resources [9]. Marginalised youth, particularly from LGBTQIA+ and culturally and linguistically diverse (CALD) groups, face even greater disparities, experiencing both limited access to inclusive resources and heightened stigma surrounding sexual health discussions [10]. Consequently, sexual health promotion among young people in Australia remains an urgent public health priority requiring inclusive strategies.

Traditional strategies for promoting sexual health among young people in Australia, primarily through school-based education and clinical interventions, still face certain limitations [11]. Current curricula are often narrowly medicalised, focus primarily on reproduction and disease prevention, and rarely address broader social and emotional aspects of sexuality, such as consent and sexual diversity [12]. These educational approaches frequently lack cultural sensitivity, failing to resonate with diverse youth audiences [12]. These limitations are further shaped by structural barriers, including heteronormativity, cultural taboos [9], distrust in official health knowledge, and a persistent digital divide [13], which together restrict young people’s access to inclusive and relevant sexual health information.

These challenges are reflected in the NSW Sexually Transmissible Infections Strategy 2022–2026 [14], which identifies sexually active young people under the age of 30 as a key priority population due to higher STI notification rates and persistent barriers to accessing care. The strategy also recognises that young people with intersecting marginalised identities—such as LGBTQIA+ communities, Aboriginal people, and those from culturally and linguistically diverse backgrounds—may be more likely to experience these barriers. In response, the strategy calls for inclusive, community-driven health promotion that leverages priority settings beyond traditional clinical spaces, including online environments. Digital platforms such as podcasts therefore represent an important opportunity to engage young people in ways that are accessible, culturally appropriate, and aligned with their everyday media use, particularly for those who may feel excluded or stigmatised in conventional healthcare settings.

Recently, podcasts have gained considerable popularity as a flexible, digital audio format that accommodates a wide range of topics, including health communication [15]. The key advantage includes convenience, with users able to listen at any time and location, including while undertaking other daily activities, making them potentially valuable tools for disseminating health information during everyday life [15]. In Australia, listenership among those aged 18–24 has risen sharply, with national surveys indicating that over two-thirds of this age group engage with podcasts monthly [16]. Podcasts are increasingly recognised as promising tools for health promotion due to their conversational tone, peer relatability, and ability to deliver complex information in digestible and engaging ways [15]. Their private and personalised format may also reduce the stigma associated with seeking sexual health information, especially on topics often avoided in traditional settings, such as schools or clinics [17].

In response to these developments, the HIV and Related Programs (HARP) Unit in the Illawarra Shoalhaven Local Health District (ISLHD), commonly known as the Caddyshack Project created the “On The Couch” podcast specifically targeted at young people, professionals who work with young people, and the broader community. The podcast places particular emphasis on reaching marginalised groups which are often underserved by mainstream sexual health promotion, including LGBTQIA+ young people, First Nations youth, young people with a disability, those from culturally and linguistically diverse (CALD) backgrounds, and professionals who work with them.. The podcast aims to adopt a casual, conversational tone, accessible and non-judgemental, and has an honest and relatable dialogue around topics of sex, sexuality and sexual health as well as current social themes [18]. Through the combined professional perspectives of experts, the podcast aims to address various topics related to the sexual lives of young people, including sexual consent, pleasure, sexual violence prevention, STIs and HIV education, contraception and protection, and navigating relationships [18]. Available widely on platforms such as Spotify, On The Couch aims to reduce the harm associated with STIs, including HIV and Hepatitis C, through partnerships with youth services, LGBTQIA+ agencies and community groups [18].

### Aim and objectives

Despite the practical implementation and growing reach of the On The Couch podcast to professions, there remains a notable research gap regarding its impact on young listeners. Existing literature primarily focuses on podcast feasibility, design, and content development without examining the experiences and perceptions of youth audiences [19,20]. Findings from a preceding scoping review [21] similarly revealed a lack of studies assessing how young people engage with podcast-based sexual health content, or whether it contributes to meaningful changes in knowledge, attitudes, behaviours, or health outcomes. Consequently, the extent to which podcast content translates into improved sexual health knowledge, attitudinal shifts, or sustained behavioural changes remains unknown.

The primary aim of this study is to explore how the On The Couch podcast supports young people’s sexual health by fostering collaborative knowledge building, reflective peer dialogue, and emotionally meaningful engagement with sexual health information to address this critical gap. Rather than measuring sustained behavioural change, this study focuses on young people’s perceived impacts, including changes in knowledge, attitudes, emotional responses, and behavioural intentions. In this study, “effectiveness” is understood as perceived effectiveness, reflecting participants’ reported impacts and appraisals of the podcast, rather than objectively measured behavioural outcomes. The study further seeks to understand how these processes vary across diverse cultural and sexual identities. Through capturing and analysing these lived experiences, this study will provide valuable insights into the effectiveness and limitations of podcast-based sexual health promotion strategies among young people in Australia.

This study involves qualitative focus groups aiming to comprehensively and deeply evaluate the effectiveness of the On The Couch podcast in promoting the sexual health of young people. The specific objectives are to:

1. Assess young people’s perceptions of the On The Couch podcast’s effectiveness in improving awareness, knowledge, behavioural attitudes, and motivations related to sexual health.
2. Explore how young people from diverse social, cultural, and sexual identities engage with various elements of the On The Couch podcast, including its format, content, duration, tone, inclusivity, and accessibility.
3. Investigate how On The Couch podcast-based content facilitates peer communication, self-reflection, and re-engagement with previously avoided or inaccessible sexual health information in a focus group setting.
4. Identify perceived limitations of the On The Couch podcast and gather participant-informed suggestions for improving the impact of podcast-based sexual health promotion.
5. Compare young people’s preferences for the On The Couch podcast and other digital media platforms such as TikTok and YouTube in the context of sexual health-related topics.
6. Explore additional health-related topics that young people would like the On The Couch podcast or similar youth-focused podcasts to cover in the future.

The insights obtained from this qualitative study will assist in refining the content, format, and delivery strategies of the On The Couch podcast to better align with the diverse needs of young audiences. They also offer practical guidance for the future development of health-related podcast interventions that can be applied in broader young people’s sexual health education and digital health promotion initiatives.

## Methods

### Study design and ethics statement

This qualitative study adhered to the Standards for Reporting Qualitative Research guidelines (SRQR) [22] (See Appendix A for the SRQR Checklist), and the design consisted of two sequential stages. In the first stage, a list of focus group questions was developed based on the scoping review findings and received ethical approval (See Appendix B for the Focus Group Questions List), which was then used to qualitatively evaluate how young people perceive and respond to the On The Couch podcast through thematic analysis. Eligibility criteria to participate in the focus group included being aged between 18 and 24 years, able to speak and understand English, and currently living in Australia.

Participants were selected according to their availability and age on the registration form. About seven days prior to a scheduled group, we sent out an invitation email to the selected participants. The email contained the date and time of the group and a hyperlink to a Qualtrics informed digital consent form (See Appendix C for the Consent Form and Participant Information Sheet).

Only those who completed the form were provided with the actual location or meeting link. Verbal consent was also obtained at the beginning of each focus group. Prior to discussion, the facilitator first spent a few minutes reiterating the same ground rules mentioned in the consent form, including key expectations such as maintaining confidentiality, respecting others’ contributions, and the right to withdraw at any time. Participants could take this opportunity to clarify their concerns. Recording via Zoom only began after all participants verbally provided their consent in the session. Ethical approval for the study was obtained from the Greater Western Human Research Ethics Committee (Reference: 2023/ETH02655), with ratification from the University of Technology Sydney Medical Research Ethics Committee.

### Focus group participant recruitment

Promotion of the study was supported by the UTS School of Public Health and the Illawarra Shoalhaven Local Health District. Initially, the focus groups were designed to be face-to-face and only held at the University of Technology Sydney [23] campus. However, we expanded the format to include online meetings via Zoom and broadened the recruitment scope to a national level. Participants were young people aged 18–24 who resided in Australia and were required to complete informed consent before participating in the study. Focus groups lasted on average 70 minutes and were conducted either face-to-face or online via Zoom; both formats used Zoom to audio record and transcribe the discussions. Participants received an AUD 30 gift card as compensation for their time and effort. Online focus group participants were asked to provide an Australian mailing address after the session to confirm their eligibility and enable gift card delivery.

In addition to more traditional methods of recruitment, such as posting announcements to students at universities, we also promoted the study through digital platforms. We created a recruitment poster with QR codes and links to a registration form developed using the online survey platform Qualtrics. To maximise visibility, the poster and registration link were distributed through targeted strategies, including university mailing lists to students, posts on LinkedIn and X (formerly Twitter), and email newsletters sent to Caddyshack subscribers and past participants of live online On The Couch events. The registration form contained a consent form and eligibility screening questions, and only those who met those criteria could leave their names (full names were not needed), age, preferred focus group format ([23] campus/online/other), and preferred contact method (email/phone number). The recruitment poster explained the backgrounds and aims of the study and listed contact methods for researchers (See Appendix D for the Focus Group Recruitment Poster). We highlighted on the poster the kind of personal information that would be collected throughout the study process, and emphasised that participation was voluntary and confidential. The recruitment period for this study was from 1 December 2024–31 January 2025. All participants provided written informed consent electronically via email prior to participation, using the ethics-approved Participant Information Sheet and Consent Form.

### Data collection

Choosing a suitable meeting and recording platform for face-to-face and online focus groups was based on a balanced decision between cost, data security, and usability. This project utilised the videoconferencing tool Zoom for online focus groups, as well as recording and transcribing both online and face-to-face focus groups.

We chose this platform mainly because:

- UTS already held a business license for its staff and students to use the program freely, so the research team did not need to search and pay for another platform [23]; the platform was also free for the public and accessible to join a meeting via personal computers or mobile devices.
- An information technology infrastructure was already established between the University and Australia’s Academic and Research Network (AARNet) to ensure online security and data privacy of Zoom recordings [24].
- As Zoom was one of the most popular meeting software in Australia during the COVID-19 pandemic [25], we suspected that most young participants would have some familiarity with the platform already.

A total of five focus groups were conducted for this study, with the first two held online via Zoom and the remaining three conducted face-to-face. To encourage participants to share sexual-related experiences comfortably in a group setting, in the first two online focus groups, participants were grouped by gender. The first group comprised female participants (n = 5), while the second consisted exclusively of male participants (n = 3). All other focus groups involved mixed-gender participants with three to four participants each. The first author (YXZ) facilitated all groups under the supervision of other authors (DD or AS) in English. Both (DD and AS) are experienced researchers and health professionals. (YXZ) is an Honours student in Public Health and belongs to the same generation as the study participants.

The online focus groups were scheduled on weekdays at 6 p.m., while the face-to-face sessions were held on weekends to accommodate participants’ availability. Prior to the discussion, the facilitator initiated casual conversations with the participants to increase their comfort and build rapport prior to the formal discussion. Each group began with the facilitator discussing the purpose and ground rules for discussion (e.g., respect each other’s comments). Focus groups used a semi-structured approach to build rapport and adapt the conversation based on the needs of the participants, including changing the order of questions and clarifying questions, as well as adding or changing questions as needed.

All online focus groups were automatically audio- and video-recorded using Zoom, while face-to-face groups were audio-recorded only, also using Zoom. Audio recordings were reviewed and cross-checked against Zoom’s automatic transcription by the first author (YXZ), and subsequently cleaned and transcribed verbatim to produce the final transcripts. All final transcripts were reviewed by the other two authors (DD and AS), after which all focus group audio recordings were permanently deleted to maximise the protection of participants’ data privacy and security.

### Data analysis

After each focus group, the facilitator conducted an informal verbal debrief with the research supervisors to reflect on the group’s process and identify potential areas for improvement in subsequent sessions. The analysis was conducted using Braun and Clarke's six-phase thematic analysis [26], which is a widely adopted and well-established method for systematically analysing qualitative data, such as interviews and focus group transcripts [27]. Each phase was methodically implemented as follows:

(i) Familiarising the researcher with the data:

The first author (YXZ) commenced the analysis by thoroughly reviewing the focus group audio recordings and verbatim transcripts multiple times to become deeply acquainted with the corpus. During this stage, preliminary reflections and recurring points were documented in an analytic note, which served as a memo to capture early thoughts, potential codes, and thematic observations during the familiarisation process.

(ii) Generating initial codes:

After the familiarisation process, the first author (YXZ) systematically generated initial codes across the entire dataset using an open coding approach grounded in inductive reasoning, allowing data-driven insights to emerge without imposing pre-existing frameworks [26]. Transcripts were examined line by line to identify and label pertinent features related to the research objectives. Coding was first conducted manually using Microsoft Word software and different colour highlighting and thematic notes to facilitate a detailed, iterative approach. Following the initial coding, all codes and interpretations were reviewed and discussed in person during meetings with the two academic supervisors, which helped ensure analytical rigour, consistency, and reflexivity in the coding process.

(iii) Searching for themes:

Initial codes were then reviewed, collated, and grouped to identify broader patterns and preliminary themes. During this stage, codes sharing conceptual similarities were organised into clusters reflecting the research objectives, resulting in an initial thematic list comprising six themes and potential sub-themes.

(iv) Reviewing themes:

The first two focus group transcripts were initially coded by the first author (YXZ) and independently reviewed by the other two authors (DD and AS). Themes emerging from the coding were systematically discussed during research team meetings, enabling iterative refinement and shared interpretation of the data. Through this collaborative process, an analytical framework was co-developed to ensure consistency and rigour in the thematic analysis across all transcripts.

The preliminary thematic list underwent a rigorous review process involving both internal and external coherence assessments. Internally, each theme and sub-theme was carefully evaluated to ensure codes fit cohesively; externally, themes were assessed to confirm they accurately correspond to each research objective. Regular research team meetings facilitated iterative discussions and decided to merge the original six themes into three that would be refined to enhance thematic clarity and coherence. This decision followed Braun and Clarke’s [26] emphasis on parsimony and conceptual clarity in theme development.

(v) Defining and naming themes:

Following the review, themes and sub-themes were further defined and clearly named to accurately encapsulate their meaning and scope. Detailed theme and sub-theme descriptions demonstrating explicit alignment with the study objectives are presented and discussed in the following section.

Producing the report:

The final phase involved integrating the themes into a coherent and meaningful narrative that went beyond surface-level description. Each theme was developed through interpretative analysis, illustrating not only what participants said but also how and why these perspectives matter in the context of podcast-based sexual health promotion. Illustrative quotes were selected to highlight the diversity and depth of participant views while remaining grounded in the data. Thematic interpretations were discussed and refined in regular team meetings, which helped confirm the coherence and credibility of the final themes. This process ensured that the final report offered a rich and theoretically informed account of young people’s experiences and perceptions.

### Researcher reflexivity and positionality

As the primary researcher, I (YXZ) acknowledge that my own cultural, educational, and personal background may have influenced the research process. I am a heterosexual male international student from China, conducting this study within an Australian university context, exploring sexual health promotion among young people. I knew I couldn’t fully understand all the backgrounds of my participants, but I made a conscious effort to listen respectfully and ask open-ended questions without assumptions.

In conducting focus groups, I was conscious of how differences in cultural background, age, and lived experience could shape the dynamics of participant disclosure, particularly on sensitive topics such as sexuality, relationships, and stigma. For instance, during one session, I noticed that a participant hesitated when discussing queer identity and only opened further after another participant shared their experience. This experience made me reflect on how my identity may have influenced the perceived safety of participants with marginalised sexual identities. These positional differences may have led some participants to moderate their responses, while others may have felt more at ease discussing topics with a researcher closer to their culture.

Throughout the thematic analysis, I engaged in ongoing reflexive journaling to critically examine how my interpretations were shaped by my perspective, which helped mitigate individual bias and reinforce analytic rigour. In my journal after the second focus group, I noted that I had unconsciously framed a follow-up question using clinical terms like ‘risk behaviours’, which may have made participants feel judged. Reflecting on the incident helped me rephrase future questions in a more open and participant-led manner. While complete objectivity is neither possible nor expected in qualitative research, I endeavoured to maintain reflexivity and transparency by acknowledging how my position may have influenced the knowledge production process.

## Results and discussion

Five focus groups were conducted with eighteen young participants from Australia who had listened to at least two episodes of the On The Couch podcast. The demographic data of the participants are shown in Table 1.

**Table 1:** Demographic characteristics of focus group participants (N = 18).

Characteristic	n	%
Gender		
Female	10	55.6
Male	8	44.4
Age group		
18–24 years (all participants)	18	100.0
Sexual orientation		
Heterosexual	13	72.2
LGBTQIA+ (e.g., queer, lesbian)	4	22.2
Not stated	1	5.6
Cultural or ethnic background		
White Australian	7	38.9
Asian background (e.g., Chinese, South Asian)	7	38.9
Other CALD background	2	11.1
Not stated	2	11.1
Education background		
University undergraduate	15	83.3
TAFE/ vocational education	2	11.1
Not enrolled/ other	1	5.6

Note: Percentages are rounded to one decimal point. Some totals may not sum to exactly 100% due to rounding. Data were self-reported or inferred from participant dialogue where relevant.

In the focus group transcription data, three core categories of themes were identified. These are: (1) Theme 1: Knowledge, awareness, and behavioural change; (2) Theme 2: Engagement with podcast features and accessibility; (3) Theme 3: Podcast critiques and suggestions for improvement.

Table 2 summarises each theme with a brief definition and an illustrative quote to demonstrate how the theme was expressed across the focus group transcripts.

**Table 2:** Overview of key themes, definitions, and illustrative quotes.

Theme	Definition	Example Quote
Theme 1: Knowledge, awareness, and behavioural change	How podcasts enhanced understanding, shifted attitudes, or motivated behavioural intentions.	“Before this, HIV was honestly something I associated with fear… I didn’t know someone living with HIV could have a baby, or be in a relationship, or live a completely healthy life.” – Participant 1, FG3
Theme 2: Engagement with podcast features and accessibility	Participant responses to podcast structure, tone, and how accessible the format was.	“I found it [the podcast] kind of formal and removed like something you’d hear in a lecture rather than a podcast made for young people. It felt like they were talking about us, but not really to us.” – Participant 2, FG5
Theme 3: Podcast critiques and suggestions for improvement	Feedback on what could be improved in content or coverage of future episodes.	“It’s better if we could give us some examples of that, like what is on this platform and how it works. So, I want her to set up some examples, but not in detail. If we could set up an example, someone is doing it, and how it works and what happens, and that would be more engaging.” – Participant 1, FG1

### Theme 1: Knowledge, awareness, and behavioural change

The first theme explores the ways in which podcast-based sexual health education affects young people’s awareness and knowledge of sexual health, promoting attitude change, and motivating healthy behaviours. These accounts primarily reflected behavioural intentions rather than confirmed and sustained behavioural change. Drawing on research objective 1 (to assess young people’s perceptions of the podcast’s effectiveness in improving awareness, knowledge, behavioural attitudes, and motivations related to sexual health) and objective 3 (to investigate how the podcast content facilitates peer communication, self-reflection, and re-engagement with previously avoided or inaccessible sexual health information in a focus group setting), five sub-themes were identified. Participants described how the podcast content encouraged peer communication and self-reflection among young people in focus group settings and helped them reconnect with previously avoided or difficult-to-access topics related to sexual health.

#### Sub-theme 1.1: Enhanced understanding and new information.

This sub-theme captures the potential of podcasts in providing young people with accessible, relevant and potentially novel information concerning sexual health. Participants described encountering key concepts such as HIV treatment, biomedical prevention, and reproductive possibilities for people living with HIV for the first time. This occurred in contrast to formal sex education content that may be partial or outdated. In this context, the podcast was not just informative but instructive, filling in knowledge gaps.

As an example, one participant reflected that *“The podcast was about HIV. It was informative and educational… It definitely broadened my understanding of how HIV is actually treated”* (Participant 2, FG1), signalling a shift to a more accurate understanding of the topic. Another participant noted the personal impact of learning that people living with HIV could lead full and relationally rich lives: *“I didn’t know someone living with HIV could have a baby, or be in a relationship, or live a completely healthy life”* (Participant 1, FG3). In this context, the information disrupted listeners’ longstanding associations of HIV with death or social exclusion.

Beyond individual knowledge acquisition, the podcast medium was also seen as a socially valuable tool for professional development and future practice. One participant, a nursing student, reflected that while the content might not change their personal habits, *“it gives me that standing for the future to not hold the stigma”* (Participant 2, FG1), indicating how new information can influence not only self-understanding but also broader professional ethics.

Participants also drew attention to the emotional dimensions of knowledge with one participant explaining that *“I wouldn’t say I learned new facts, but my understanding of the emotional and social layers around sexual health deepened”* (Participant 4, FG5), suggesting that learning was not limited to biomedical facts but extended to affective and interpersonal insight — aspects that may be overlooked in conventional education. Others expressed scepticism about the limits of information alone in changing behaviour, with one stating, *“just giving people information doesn’t always lead to change”* (Participant 2, FG5), emphasising the need for emotional resonance and practical pathways alongside knowledge.

#### Sub-theme 1.2: Attitudinal changes and perception shift.

This sub-theme highlights how the podcast’s use of real-life stories and emotional resonance encouraged participants to reassess their attitudes and understanding of sexual health. By centring lived experiences, the podcast offered a more humanising lens on topics that are often presented through fear-based messaging in formal education.

The storytelling approach was perceived to be better at connecting with participants emotionally than formal or academic language, leading to positive changes in their attitudes and encouraging them to rethink their existing stereotypes and biases about sexual health. As one participant expressed, *“Tasha’s story about motherhood and HIV — it hit me in a way that no sex ed class ever did. Like, that’s what I want from a podcast — real voices, not just experts”* (Participant 3, FG3). This highlights the significance of authenticity and relatability in health education, where lived experience can challenge dominant narratives and evoke critical reflection. Similarly, another participant noted that *“hearing someone like Tasha talk about her experience — that’s different. That is my world”* (Participant 3, FG3), suggesting that resonance is rooted in perceived proximity and shared reality.

These attitudinal shifts were not limited to emotional recognition but extended into cognitive reappraisal. Several participants acknowledged that the stories disrupted longstanding stigmas and moralistic framings of HIV. One participant reflected, *“Before this, HIV was honestly something I associated with fear. It was talked about church as a punishment or something only ‘immoral’ people get, which I know now is totally wrong”* (Participant 1, FG3), underscoring the de-othering effect of personal storytelling. The shift from fear and judgment to empathy and normalisation is consistent with sociological theories of stigma reduction, which emphasise the role of interpersonal exposure and narrative in reshaping public perceptions.

#### Sub-theme 1.3: Behavioural intentions and motivational impact.

This sub-theme explores how podcast-based content prompted participants to consider taking action related to their sexual health. While not all listeners reported immediate behavioural changes, many described feeling more motivated to seek testing, initiate conversations, or reflect on future professional practice. The podcast was perceived as lowering emotional and social barriers to these behaviours by presenting information in relatable, non-judgmental ways.

Some participants described how the content led them to contemplate real-world actions. For example, one shared, *“For me, it was more than just reflection. I actually looked up where to get tested near my uni. I haven’t booked it yet, but that’s already something I wouldn’t have done before”* (Participant 1, FG3), suggesting that the podcast functioned as a behavioural prompt. Rather than prescribing action, the conversational tone created space for self-directed decision-making. This illustrates how podcast content can operate as a “soft prompt” within the behavioural intention stage of the Theory of Planned Behaviour, particularly by lowering the perceived social cost of action in contexts where sexual health remains taboo.

For those studying or working in health, the podcast also influenced professional aspirations. A nursing student explained, *“I believe it would impact how I actually practice as a nurse… It gives me that standing for the future to not hold the stigma”* (Participant 2, FG1), indicating how motivational impact extended beyond personal health to ethical positioning in care settings.

Others noted that the podcast gave them practical ideas for how to initiate conversations about sexual health. A pair of participants commented that *“So maybe not full-on action yet, but I’d call it movement. And honestly, I brought it up with Participant 2 last night too like, whether we’ve been avoiding certain conversations out of habit or awkwardness”* (Participant 1, FG4), highlighting how podcasts may serve as social scripts or behavioural rehearsals. However, some remained cautious about equating motivation with behaviour. As one put it, *“I wouldn’t say it changed my behaviour but it did make me think more compassionately about others who haven’t taken that step yet”* (Participant 4, FG5), underscoring that knowledge and motivation are only part of a complex pathway to action. Overall, participants saw podcasts as a valuable first step, one that promotes readiness and reduces stigma, even if action is delayed. Notably, while participants articulated a readiness to act, their narratives suggest a delayed enactment, highlighting a potential conflict between intention and behaviour often overlooked in linear health behaviour models.

#### Sub-theme 1.4: Emotional impact vs. behavioural sustainability.

This sub-theme reflects how participants were often emotionally affected by the podcast, especially by personal stories shared by the guests. While some participants described moments of personal reflection on their own attitudes or past experiences, these emotional reactions did not always lead to sustained behavioural change. The podcast served as a prompt for thinking but not always as a catalyst for action. These emotional reactions can be understood not only as personal reflections but also as affective responses to dominant discourses of risk, morality and health responsibility embedded within sexual health communication and education.

Participants frequently described being “hit emotionally” by the content, particularly through authentic, lived experiences that contrasted with the detached tone of traditional sex education. One noted that the episode *“It felt a bit like a wake-up call. Not in a panic kind of way, but in a why haven’t I done this before?”* (Participant 1, FG4), indicating that emotional intensity fostered connection. While these emotional reactions were powerful, participants often struggled to translate them into concrete actions or decisions. As one participant put it, *“You might know something but still not feel like you can act on it”* (Participant 2, FG5), highlighting a gap between internal response and external change.

Some participants viewed emotional impact as a necessary but insufficient catalyst. A comment from FG5 captured this tension: *“Podcast can spark interest but people need follow-up or somewhere to go after that. Otherwise, it just stays as content and doesn’t really move people”* (Participant 4, FG5), reflecting the concern that affective learning alone cannot sustain behaviour without structural reinforcement [28]. This also aligns with the notion of affective publics [29], where emotional engagement does not inherently translate into political or behavioural outcomes without community or institutional support. Others added that they appreciated the emotional tone but were already familiar with the content, which limited its transformative potential. For instance, one shared, *“I’m glad I know the information, but I think for some of them I already knew, and I guess it won’t change my way of [thinking]”* (Participant 1, FG2).

This disconnection matches important theories about changing behaviour, like the Health Belief Model (HBM) and Social Cognitive Theory (SCT), which both say that just feeling strong emotions isn’t enough to keep someone changing their behaviour. According to HBM, individuals are unlikely to act solely based on emotional reactions unless they also perceive themselves as susceptible to risk, believe in the severity of consequences, and receive clear cues to action and reassurance about potential barriers. In parallel, SCT emphasises that emotional arousal may generate awareness, but it must be accompanied by self-efficacy, observational learning, and supportive environments to lead to meaningful change. As Bandura [30] noted, emotional responses need to be paired with a sense of control and actionable pathways; otherwise, motivation may remain dormant. The participants’ reflections in this sub-theme exemplify this theoretical gap: they were moved but lacked the tools or confidence to act.

#### Sub-theme 1.5: Cultural influences on sexual health awareness, knowledge, and behaviours.

This sub-theme highlights how cultural and religious norms shape young people’s ability to engage with sexual health information, often contributing to silence, stigma, and constrained behavioural responses. Across the focus groups, participants reflected on how deeply embedded values from family, religion, and peer contexts limit open dialogue of sexual health knowledge.

Several participants described growing up in environments where sexual health was considered taboo, with one explaining that *“In my culture, we rarely discuss sexual health issues or related topics”* (Participant 1, FG1). These silences were not merely interpersonal but perceived as moral imperatives shaped by religion. For instance, one participant reflected on the guilt instilled by their upbringing: *“Something related to this is that even all the social media platforms banned us from watching porn or getting any knowledge of how sexual life is meant to be”* (Participant 1, FG1), illustrating how internalised stigma continues to restrict engagement, even when accurate information is available. Notably, although participants often described the cultural and religious stigma surrounding sexual health, the specific podcast episodes included in this study did not explicitly engage with cultural or religious norms as barriers to sexual health communication. This reflects a limitation of the specific podcast episodes analysed in this study, rather than of the whole Caddyshack podcast project or of the study design, as the present study examined young people’s responses to an existing podcast intervention and did not involve co-designing content tailored to specific cultural contexts. Accordingly, participants’ accounts highlight a mismatch between the broadly inclusive messaging of the selected Caddyshack episodes and the more context-specific moral and cultural constraints faced by some young people, particularly those from religiously conservative backgrounds.

Podcast exposure was sometimes described as a catalyst that gently disrupted these inherited taboos. A participant shared that *“Hearing people speak so openly kind of made me feel like maybe it’s not something to be ashamed of”* (Participant 1, FG5), suggesting that relatable audio storytelling may help reframe sexuality as a topic worthy of discussion. However, others expressed that while the podcast prompted reflection, cultural constraints continued to inhibit behaviour change, particularly when broader social or family norms were unsupportive. One participant shared, *“In my family, and honestly in a lot of households from my community, we just don’t talk about sexual health. Like, ever. Even saying the word sex in front of your parents would be out of line. It’s not even just embarrassment; it’s that it feels morally wrong, like you’re going against your faith”* (Participant 1, FG5), illustrating how family and community cultural stigma may override even strong individual awareness.

These reflections highlight that sexual health promotion for young people must be not only informational but also culturally attuned. Participants from CALD backgrounds, particularly those with religious and culturally conservative upbringings, shared how moral scripts from family and faith traditions continued to suppress sexual health promotion, even when they personally recognised the value of the podcast content. This illustrates a tension: people may receive and intellectually accept sexual health knowledge, but when it conflicts with deeper cultural norms, it becomes behaviourally inert. The podcast’s inclusive tone created emotional impact, yet its limited direct engagement with cultural and religious constraints may have reduced its relevance and practical utility for some listeners. From a HBM perspective, even when young people perceived the benefits of sexual health information, the absence of culturally sensitive cues for action and the presence of strong barriers, such as shame or familial disapproval, interfered with behaviour change. Likewise, SCT suggests that emotional arousal must be accompanied by self-efficacy and enabling environments to drive sustained action, factors largely absent in these accounts. This points to an important implication for future podcast development: culturally responsive content (e.g., explicitly acknowledging faith-based or culturally grounded concerns) may better support young people who experience these constraints. This theme also matches the results from the study, which found that there aren’t enough culturally relevant frameworks in current podcast-based programs for CALD youth [31].

### Theme 2: Engagement with podcast features and accessibility

The second theme examines how specific features of the podcast, such as tone, format, inclusivity, and technical accessibility, influenced young people’s engagement with its content and perceived effectiveness as a sexual health communication tool. Drawing on research objectives 2 (to explore how young people from diverse social, cultural, and sexual identities engage with various elements of the podcast, such as its format, tone, inclusivity, and accessibility) and 5 (to compare the podcast with other digital media platforms like TikTok and YouTube in the context of sexual health communication), four sub-themes emerged from the data, reflecting how participants evaluated the podcast’s delivery style, navigated its technical affordances, and compared it to other digital media they routinely used. Participants described how these elements affected their willingness to listen, their ability to retain information, and their emotional connection to the material.

#### Sub-theme 2.1: Podcast format and content delivery.

This sub-theme explores how the podcast’s format, particularly its conversational tone, narrative style, and use of diverse voices, shaped young people’s engagement with the content. Participants frequently emphasised that these stylistic features were central to the podcast’s perceived accessibility, emotional resonance, and educational value. By moving away from traditional didactic formats, the podcast was seen to foster relatability, trust, and multidimensional understanding.

Several participants praised the use of natural and discussion-based delivery, highlighting that *“I like how you can just dive into the conversation in a way that feels engaging and fluid”* (Participant 3, FG1). This conversational style was repeatedly associated with increased clarity and emotional connection, particularly among those who found formal education settings alienating. The inclusion of multiple speakers and personal stories further enhanced this effect. For instance, one participant noted, *“I also really like casual, back-and-forth style conversations — like when people bounce ideas off each other. That makes it feel more real, less scripted”* (Participant 3, FG4), indicating that the interactive structure contributed to a stronger sense of authenticity. The strong preference for unscripted, peer-like delivery can be seen as preferencing experiential over credentialed authority by young people.

Others valued the inclusive tone of the podcast, explaining that its respectful and non-judgmental approach supported a sense of psychological safety. As one participant expressed, *“So by sharing their own experience, it helps them to understand each other and also find certainty”* (Participant 1, FG1), suggesting that stylistic delivery can mediate power dynamics and improve receptivity to sexual health messages. Conversely, some participants critiqued the overly professional or academic tone in certain episodes, which *“sounded like a lecture” and “don’t feel the connection”* (Participant 3, FG3), revealing a tension between perceived expertise and engagement. This suggests that future episodes of the On The Couch podcast targeting young people specifically should avoid overly clinical or academic tones. Participants consistently responded more positively to content that felt authentic, personal, and peer-led, rather than expert-dominated. This critique shows how perceived legitimacy in health messaging is increasingly tied to narrative authenticity rather than formal expertise, a shift potentially also reflective of broader sociocultural distrust in institutional authority, particularly around health and wellbeing.

Young participants consistently associated the podcast’s conversational format with greater engagement, emotional resonance, and informational clarity. This preference highlights that delivery style shapes how information is emotionally processed and cognitively retained, especially when the narrative feels familiar and non-authoritative. Youth voices, storytelling, and informal pacing helped reduce perceived power imbalances and foster a sense of shared experience, making the content easier to absorb. While most participants responded positively to this relaxed style, others critiqued episodes that felt “too professional” or “like a psychology lecture”, revealing diverse expectations shaped by differing educational backgrounds and prior exposure to health communication. These reflections align with Objective 2 by illustrating how young people engaged with podcast-specific elements such as tone and format, and with Objective 5 by clarifying why they often preferred the On The Couch podcast over other platforms like YouTube or TikTok, which were considered either too fragmented or too entertainment-focused. From a Social Cognitive Theory perspective, these findings underscore the power of modelling: hearing peers openly discuss sexual health helped build observational learning and emotional trust, both critical precursors to behaviour change. Without that peer-based credibility, motivation may falter even if the information is technically accurate. This interpretation is consistent with the study [32], which identified that youth-oriented sexual health podcasts are most effective when they feature diverse voices, conversational structure, and peer-relevant narratives that reduce psychological distance.

#### Sub-theme 2.2: Accessibility and technical ease.

This sub-theme highlights how the technical ease and flexible access afforded by podcasts shaped participants’ engagement with sexual health content, particularly in comparison to more formal or screen-based educational formats. The ability to listen on-demand and in varied environments made the medium particularly attractive to young people managing busy or multitasking routines.

Participants commonly described the podcast format as seamlessly integrated into their everyday lives, offering opportunities to absorb information while engaging in other tasks. For instance, one participant noted that they *“could use it at any time when I’m cooking, taking a shower, or just on my commute way back from home”* (Participant 1, FG1), suggesting that the audio-only nature allowed sexual health content to be consumed passively yet meaningfully. Similarly, listening during commuting or while doing chores was seen as an efficient and low-effort form of health education, underscoring the affordance of temporal flexibility and cognitive multitasking.

However, others drew attention to the limitations posed by the lack of visual and textual support, especially for those who rely on visual reinforcement or subtitles for better comprehension. One participant reflected, *“If it doesn’t show up visually with a caption or a quote no one’s clicking through”* (Participant 2, FG5), indicating that engagement was partially dependent on individual learning styles and accessibility needs. The absence of supplementary visual cues was also noted as a potential barrier for audiences with hearing impairments. This potential technological unawareness also signals a potential class-based or linguistic divide in accessing health knowledge, raising equity concerns about who benefits from inclusive digital formats that are aimed at inclusivity.

These diverse responses suggest that while podcast-based learning offers convenience and accessibility, it may not meet all users’ needs equally. Suggestions such as incorporating visual infographics, or live captions were raised to enhance inclusivity and comprehension. As one participant proposed, *“Subtitles or a transcript would help me keep up”* (Participant 1, FG3), highlighting a desire for more multimodal engagement options. Interestingly, some of these features, such as auto-generated transcripts, are already available on mainstream platforms like Apple Podcasts; participants seemed unaware of their existence. This suggests that accessibility gaps may stem not only from technological absence but also from low visibility. Participants’ calls for clearer summaries also point to the need for structural design choices within podcast episodes themselves, such as verbal recaps or key takeaways at the end of each episode, to support different learning preferences. Although it should be noted that transcripts are currently available, some participants may not have been aware of them or may have expected them to be presented in a more accessible or visible format.

This sub-theme shows that podcast accessibility is not just about whether the content is available, but about whether it meets the needs of different types of listeners. Participants valued the ability to listen while multitasking, but their responses also showed that access and engagement depended on how they processed information. Many required visual reinforcement, captions, or clearer summaries to fully understand the content. The Health Belief Model (HBM) suggests that without strong cues to action [33], such as visual prompts or verbal recaps, motivation to act on the information may remain low. Interestingly, some accessibility tools, such as auto-generated transcripts, already exist on major platforms, but participants were unaware of them. This highlights a gap between technical availability and user awareness. These findings show that while podcasts can be flexible and accessible, truly inclusive design must account for different learning needs, attention styles, and ways of retaining information beyond audio alone.

#### Sub-theme 2.3: Length and attention span.

This sub-theme explores how the podcast’s duration influenced young people’s willingness to engage, sustain attention, and perceive informational value. Participants consistently reflected on the importance of matching length to attention capacity, signalling a broader challenge for long-form audio content in capturing and retaining youth engagement. While some valued the opportunity for in-depth discussion, others expressed frustration when the content felt overly extended or insufficiently structured.

Several participants described the 30-minute duration as a functional upper limit for concentration and knowledge retention, with one noting that *“anything longer than half an hour, and I just zone out — especially if I’m not doing something while listening”* (Participant 1, FG2). This reflects a broader trend among digital-native audiences, where shorter content is often perceived as more usable and compatible with fragmented schedules. Another participant explained, *“If it’s too long and doesn’t move, I stop paying attention. There’s just so much else I could be doing”* (Participant 2, FG4), suggesting that lack of content momentum can directly lead to disengagement.

However, others emphasised that longer formats could be effective when paired with compelling storytelling or expert insights. One participant remarked, *“I don’t mind 40 minutes if it’s interesting and not just repeating stuff. But if it drags, I’m out”* (Participant 3, FG5), indicating that perceived value, rather than absolute length, is what governs sustained engagement. These perspectives underscore the challenge of balancing depth with digestibility, particularly in health promotion formats.

This sub-theme reveals that podcast length alone does not determine engagement; rather, it interacts with pacing, perceived value, and content relevance. Participants repeatedly stressed that episodes exceeding 30 minutes risked losing their attention unless the content remained dynamic and emotionally engaging. While some preferred shorter formats due to limited attention spans shaped by platforms like TikTok and YouTube, others were willing to invest more time when the topic felt meaningful or when delivery included vivid storytelling, practical relevance, or peer voices. These findings align by illustrating how participants engaged with specific podcast elements like duration and narrative structure and revealing why short-form content remains dominant among youth. According to Social Cognitive Theory, attention and retention are enhanced when observational learning is supported by emotional resonance. Participants’ engagement increased when the podcast incorporated these elements, suggesting that sustained attention is not merely a function of time but of content and emotional connection. This interpretation is supported by the study, which found that youth-focused health podcasts were most effective when they delivered focused content in well-paced formats that matched the attention habits of digital-native audiences [31].

#### Sub-theme 2.4: Comparisons and preferences with short-form medium.

This sub-theme captures participants’ comparisons between podcast content and short-form media platforms such as TikTok and YouTube, highlighting how format, interactivity, and immediacy shape young people’s media preferences when engaging with sexual health topics. While podcasts were recognised for their narrative depth and reflective potential, short-form formats were often perceived as more aligned with youth’s attention patterns and digital consumption habits.

Participants repeatedly described short videos as more compatible with fast-paced lifestyles, suggesting that immediacy and brevity were essential for maintaining engagement. As one participant explained, *“If it was on TikTok, I would probably watch it more — short, sharp, and grabs your attention”* (Participant 2, FG3), indicating that platform familiarity and design affordances contribute to higher exposure. Another noted the interactive appeal of short-form platforms: *“TikTok has comments and stitches — you can actually see other people reacting, which makes it feel more alive”* (Participant 3, FG1), suggesting that peer-driven content may foster a greater sense of community involvement and responsiveness than passive listening alone.

This preference was not universally endorsed. Some participants appreciated the depth podcasts offered, particularly for complex or emotionally sensitive topics that required more context. One remarked, *“Short videos are good for a quick message, but sometimes you need more than 30 seconds to get what someone’s really saying”* (Participant 1, FG5), reflecting a perceived trade-off between emotional nuance and brevity. Others proposed a hybrid approach, suggesting that short-form content could be used to “hook interest” while podcasts provide fuller explorations.

While participants acknowledged the immediacy and shareability of short-form platforms like TikTok, they did not suggest these formats could replace the depth provided by longer podcast episodes. Rather, they described a complementary strategy, where short videos might spark initial interest but require follow-up through more reflective, narrative-rich content. However, this hybrid approach carries the risk of trivialisation if short-form content is not intentionally designed to connect meaningfully with substantive discussions. Participants indicated that content sustaining longer engagement needed to feel authentic and emotionally relevant when addressing complex issues like consent, identity, or stigma. These patterns reflect core principles of social cognitive theory, where attention is enhanced when learners encounter content that feels socially relatable and emotionally engaging. Peer-led storytelling, moments of vulnerability, and cultural specificity were repeatedly mentioned as factors that encouraged sustained attention. These reflections show how young people select and switch between media platforms based on content resonance and clarify what features (e.g., interactivity, emotional tone) make podcast-based content more engaging. Ultimately, content format alone is not enough; what matters is how well it aligns with young people’s social realities and informational needs.

### Theme 3: Podcast critiques and suggestions for improvement

The third theme captures how young people critically reflected on the limitations of the On The Couch podcast and articulated suggestions for its improvement, including future content directions. Drawing on research objective 4 (to identify perceived limitations of the On The Couch podcast and gather participant-informed suggestions for improving the impact of podcast-based sexual health promotion) and objective 6 (to explore additional health-related topics that young people would like the podcast or similar youth-focused platforms to cover in the future), three sub-themes were identified. Participants raised concerns about structural and stylistic features of the podcast and offered concrete recommendations to enhance its engagement, reach, and inclusivity. They also proposed a range of future topics they viewed as urgent, under-represented, or more reflective of their lived experiences, highlighting a desire for more expansive, intersectional, and culturally responsive approaches to podcast-based sexual health promotion.

#### Sub-theme 3.1: Perceived limitations of podcasts.

This sub-theme captures participants’ reflections on structural, stylistic, and contextual limitations that reduced the perceived impact and accessibility of the On The Couch podcast. While many recognised the podcast’s potential, several participants found that certain delivery features diminished their engagement, including slow pacing, lack of concrete examples, and overly clinical language. These critiques highlight a mismatch between how the podcast communicated its content and what young people found engaging or accessible.

Some participants expressed frustration at the podcast’s introductory segments, describing them as *“dragging on for too long”* or *“delaying the actual content,”* which led to early disengagement. One participant noted, *“I zoned out in the first few minutes… it was too much preamble before getting to the story”* (Participant 1, FG2), suggesting that attention could be lost before the key message was delivered. Others highlighted that the pacing and tone lacked dynamism, with one describing it as *“just someone talking for too long, with no shift in energy”* (Participant 4, FG5), indicating a perceived mismatch with the faster, more interactive formats young people often consume.

In addition to structural concerns, some participants found the language used in the podcast episodes listened to for the study to be *“too academic”* or *“sounding like a lecture,”* limiting relatability and emotional connection. This perception was amplified by a lack of illustrative case examples or real-world scenarios. As one participant remarked, *“They talked about stigma but didn’t really show what it looks like. I needed a real person’s story”* (Participant 3, FG2), underscoring a need for greater contextual grounding. Such comments reflect the limitations of abstracted health discourse, where stigma is often named but not socially situated.

This sub-theme highlights how structural and stylistic features of the On The Couch podcast, such as pacing, tone, and language, shaped participants’ engagement and perceived relevance. While many acknowledged the podcast’s intent, several participants described its delivery as overly academic, too slow, or lacking emotional variation. These critiques reflect more than surface preferences; they signal a deeper disconnect between traditional health communication models and the expectations of young audiences. According to social cognitive theory, attention and message retention improve when content is socially and emotionally relatable. However, participants reported disengaging during prolonged introductions or monotone narration, indicating that without narrative momentum or variation, content may fail to sustain cognitive focus. The absence of concrete case examples further reduced opportunities for observational learning and emotional connection, which are both critical for behavioural impact. As one participant noted, “*Talking about stigma isn’t enough—I needed a real person’s story*,” emphasising the need for contextual grounding. From a Health Belief Model perspective, if young people cannot recognise personal relevance or emotional salience, their motivation to act may remain low. These findings highlight that effective podcast design must integrate not only accurate information but also delivery strategies that resonate with youth media habits, emotional engagement patterns, and attention styles. Engagement appears essential for achieving meaningful impact in podcast-based sexual health promotion.

#### Sub-theme 3.2: Participant-driven improvement suggestions.

This sub-theme highlights participants’ suggestions for enhancing the impact, relatability, and inclusivity of podcast-based sexual health education. Rather than passively receiving content, many participants took an active evaluative stance, envisioning improvements that could foster stronger engagement, emotional resonance, and youth-centred design.

A recurrent theme was the desire for more interactive and dialogical formats. One participant proposed involving young people directly in the podcast, noting that *“having someone our age as a co-host or like a guest every now and then… that would make it feel less like we’re being taught, more like we’re being talked with”* (Participant 2, FG1). This underscores the value of peer voice integration, suggesting a shift from expert-dominant delivery toward co-produced knowledge environments that are more horizontal and participatory.

Other participants emphasised the need for greater authenticity through lived experience. For example, one remarked, *“I think real stories would go a long way — maybe not just professionals talking, but like people who’ve been through this”* (Participant 4, FG2), indicating that health information gains credibility and emotional impact when grounded in personal narratives. Similarly, Participant 3 in FG5 suggested that *“more rawness”* was needed *“stuff that doesn’t sound rehearsed or perfect. Like actual struggles.”* These comments reveal an appetite for emotional realism, where imperfection is not a flaw but a marker of trustworthiness and relatability.

Participants also highlighted production elements as a site for improvement. While some of these suggestions, such as adding transcripts or visual supports, were discussed earlier in relation to accessibility, they were reiterated here as part of broader calls for improved production quality. *“Better editing, some music, even chapter markers can help maintain engagement”* (Participant 2, FG3), one suggested, pointing to the potential of aesthetic and structural refinements in sustaining attention. Others once again emphasised the value of visual or transcript supplements, with one noting, *“sometimes I just need to see the words”* (Participant 1, FG4), especially for audiences with diverse language backgrounds.

These participant-driven suggestions reveal more than stylistic preferences. They point to a deeper expectation that sexual health communication should be socially engaging, emotionally relatable, and tailored to the ways young people prefer to learn. From the perspective of social cognitive theory, such responses can be understood through mechanisms like attention, motivation, and observational learning. Participants were more engaged when they saw or heard from people similar to themselves, particularly when content was delivered in a tone that felt authentic rather than instructional. Suggestions to include young co-hosts or real-life stories reflect a desire for peer modelling, where trust and credibility are built through shared identity and emotional realism. Likewise, calls for more dynamic production elements, such as music or improved editing, reflect an effort to capture attention and enhance content retention. These findings suggest that podcast-based health interventions must move beyond simply delivering accurate information, and instead consider how delivery strategies influence engagement and learning outcomes.

#### Sub-theme 3.3: Future topics for health-related podcast content.

This sub-theme captures participants’ suggestions for expanding the thematic scope of the podcast to better reflect their holistic health concerns. Although most participants had only listened to two episodes focused on sexual health, many expressed curiosity about how the podcast might evolve to include other aspects of wellbeing. Rather than criticising the existing content, which was generally well received, they envisioned future episodes that could address intersecting issues, such as mental health, reproductive justice, and identity-related challenges. This desire to “broaden” content appears grounded not in dissatisfaction with the current focus but in a recognition that youth health needs are multifaceted and often interconnected.

Mental health was the most frequently mentioned topic, often discussed with urgency and emotional resonance. One participant explained, *“I immediately think of mental health, especially things like anxiety, burnout, and identity crisis—the kind of stuff people go through in uni but don’t always talk about out loud”* (Participant 3, FG5), indicating both the relevance and silence surrounding these experiences. Others echoed this sentiment, with a participant noting that *“mental health is so linked with sexual health anyway, like how you feel about yourself, shame, confidence, all that”* (Participant 1, FG3), suggesting an integrated approach to health education.

Participants also identified child health as an area with unmet information needs. One participant observed, *“I feel like no one talks to young people about parenting or how to raise kids, but lots of us have younger siblings or even think about having kids early”* (Participant 2, FG1), pointing to a generational gap in practical health education. Another participant proposed including *“real parents talking about how they talk to their kids about this stuff”* (Participant 4, FG2), emphasising intergenerational dialogue and lived experience.

While these suggestions varied in focus, they consistently called for content that is emotionally honest, socially relevant, and grounded in everyday realities. Some participants stressed that health topics should reflect the pressures they actually face, not what institutions assume they need. As one put it, *“don’t just do what schools do—talk about the things we actually think about”* (Participant 3, FG5), underscoring a gap between health knowledge and lived youth experiences.

These findings suggest that young people view health not as a series of isolated domains but as a set of interconnected experiences shaped by emotion, identity, and context. The desire to include topics such as mental health, parenting, and intergenerational relationships reflects a shift toward more holistic and socially embedded understandings of wellbeing. From a social cognitive theory perspective, these preferences reinforce the importance of emotional resonance and contextual relevance in health communication. When participants requested “real stories” and content “we actually think about,” they were calling for podcasts to reflect their social environments and everyday struggles. This supports themes where engagement was tied not only to format and tone but also to perceived authenticity and cultural relevance. Expanding podcast content in response to these expectations may strengthen both attention and behavioural impact by the lived realities and cognitive frameworks of youth audiences.

## Summary of key findings

This qualitative study explored the effectiveness of podcasts, specifically the On The Couch podcast, in improving sexual health among young people in Australia aged 18–24. Using thematic analysis, the study examined how the On The Couch podcast supports young people’s sexual health by fostering collaborative knowledge building, reflective peer dialogue, and emotionally meaningful engagement with sexual health information. Findings were structured into three themes, each interpreted through participants’ experiences and perceptions of podcast-based sexual health promotion. This summary synthesises key insights to demonstrate how podcasts can support youth sexual health promotion and the factors influencing their effectiveness.

The study offers new perspectives by illustrating that effective sexual health promotion among young people must engage deeply with their lived social realities, emotional experiences, and cultural contexts. The adoption of the social cognitive theory (SCT) revealed that participants valued podcasts not only as a promotion tool but also as socially embedded narratives that reflected their identities, relationships, and emotional struggles. SCT emphasises that effective behavioural learning requires more than just factual health information; it also necessitates emotionally resonant narratives and peer models. Through this theoretical framing, participants’ reflections were interpreted as evidence of observational learning, emotional identification, and perceived social relevance, which helped explain why podcasts were experienced as more engaging than traditional sexual health education. From the perspective of young listeners, traditional sexual health promotion was often described as detached, overly clinical, or moralistic, qualities that failed to evoke trust or motivation. In contrast, podcasts filled this gap by integrating genuine peer voices and stories reflecting participants’ actual social and emotional environments, thereby increasing engagement and cognitive retention.

However, SCT alone cannot fully explain why emotional resonance and peer narratives do not always translate into sustained behavioural change. To account for this disconnect between emotional engagement and practical action, the Health Belief Model (HBM) provided a complementary framework. It highlighted that perceived benefits and intentions are often constrained by deeply rooted contextual barriers, such as cultural stigma, religious conservatism, and familial silence, which many participants described as unyielding despite increased sexual health awareness. Participants’ accounts were therefore interpreted not as contradictions, but as reflections of competing cultural forces shaping health behaviour. These constraints were especially pronounced among marginalised youth, including LGBTQIA+ and culturally diverse communities, whose lived environments often conflicted with the podcast’s inclusive messages. Thus, while SCT illuminated how young people connect cognitively and emotionally with content, HBM clarified the structural and cultural limitations that prevent intention from becoming action. Together, they reveal a layered understanding of the gap between awareness and behaviour.

In summary, these findings argue for podcast interventions that are designed with and for young people, which means these interpretations position young people not simply as recipients of information, but they must be grounded in the lived experiences, identities, and communication preferences of youth audiences and that they should engage young people as co-creators rather than merely treating them as a target audience. The unique contribution of this study lies in demonstrating that effective youth-focused digital health promotion requires an integrated theoretical approach. Podcast interventions should go beyond disseminating information or boosting motivation; they must also strategically address the broader cultural and environmental contexts shaping young people’s health behaviours, such as fostering emotional safety, cultural relevance, and identity reflection. By emphasising cultural sensitivity and participatory design processes that involve young people themselves, future podcast interventions can more effectively shift not only knowledge and attitudes but also the broader conditions that enable behavioural change. This comprehensive understanding reframes young people not as passive recipients of health messaging but as active social agents whose decisions are embedded in emotional, relational, and cultural dynamics.

## Methodological reflections and limitations

This study has several limitations that warrant reflection. First, the sample size was small (n = 18), and while this is common and appropriate for qualitative focus group research, it may limit the findings beyond the specific contexts represented. The sample was drawn primarily from urban areas in New South Wales, and many of the participants were university students, which may limit the transferability of findings to rural, remote, or culturally and linguistically diverse youth populations. Accordingly, the themes identified should be interpreted as context-specific rather than generalisable to all young people. While demographic diversity was considered during recruitment, the study does not claim representativeness. Rather than providing population-level estimates or causal claims, this study offers qualitative insights into how this group of young people perceived and made meaning of podcast-based sexual health content.

As with all focus group research, group dynamics may have shaped participant responses [34]. Sensitive topics such as stigma, sexuality, and identity may have been underexplored due to social desirability or peer influence, particularly in mixed-gender or unfamiliar settings [35]. In addition, researcher positionality and interpretive lens are inherent in qualitative analysis. The researchers’ assumptions and perspectives inevitably influenced the thematic development, even though they used reflexive practices [36]. Findings should be interpreted as reflecting processes of attitudinal shifts and behavioural intention formation, rather than evidence of actual or sustained behaviour change. Finally, language and cultural background may have influenced how some participants expressed nuanced views [37], indicating the importance of culturally sensitive and linguistically accessible research design in future studies.

## Future research directions

Future studies could build on these findings by exploring the long-term impacts of podcast-based interventions on young people’s sexual health behaviours. Given that the present findings are derived from a small, context-specific sample (n = 18), future research would benefit from designs that can assess whether the perceived shifts into sustained behavioural change across more diverse groups. Longitudinal or mixed-method designs would be particularly valuable in assessing whether attitudinal shifts and intentions reported in focus groups translate into sustained behavioural change.

In addition, future research should examine how integrating podcasts with visual media, such as captioned videos or social media highlights, affects accessibility, comprehension, and engagement, particularly for users with low literacy or different learning preferences, to support inclusive health communication and assist implementation efforts by local services, such as NSW Health. Given the diversity of youth experiences, there is a pressing need to develop culturally tailored podcast content for under-represented groups, including LGBTQIA+ communities, rural populations, and young people from minority ethnic backgrounds. Purposeful sampling strategies that prioritise under-represented groups may help strengthen future findings by ensuring a broader representation of young people.

Future research could also incorporate analytic data collected directly from podcast platforms, such as listener demographics, engagement metrics, and listening duration. Comparing these data analytics between young and professional audiences could provide insights into how different groups engage with the podcast and which content or formats best meet their needs.

Finally, future projects could prioritise youth co-creation, involving young people not only as target audiences but also as collaborators in designing, scripting, and testing podcast content. In particular, researchers should investigate what types of content young people find both relevant and engaging, such as personal storytelling, peer conversations, or scenario-based learning. This includes exploring how different content formats support emotional connections, knowledge retention, and motivation for behaviour change. Drawing on SCT, future studies could examine how relatable role models and emotionally resonant formats support learning and motivation. HBM can guide research into why even engaging podcast content may not lead to behavioural change among young people due to cultural or familial constraints. By extending the evidence base beyond exploratory qualitative insights through longitudinal and mixed-method designs, future research can further examine how podcasts are perceived to support sexual health promotion in diverse settings.

## Practical implications for podcast developers

In addition to guiding future research, these findings provide several practical recommendations for developing youth-focused sexual health podcasts. Podcast developers should prioritise cultural relevance and emotional authenticity, incorporating personal stories, peer-to-peer dialogues, and diverse perspectives that closely align with young people’s lived experiences. Additionally, incorporating accessibility features such as transcripts, subtitles, or visual materials can enhance engagement, particularly for audiences with varying literacy levels and learning preferences. Involving young people in the co-design of podcast content can further improve relevance, trust, and audience reach. It is also crucial to design content with sensitivity to the cultural and familial contexts that influence sexual health decisions, especially among marginalised groups. By aligning podcast content with both theory and the social realities of young audiences, practitioners can improve the likelihood that podcasts motivate young people to adopt and maintain positive health behaviours.

## Conclusion

This qualitative study provides in-depth insight into how young people engage with podcast-based sexual health promotion, highlighting their preferences, critiques, and aspirations for future content. Through five diverse focus groups, participants articulated how podcasts may support knowledge development, prompt attitudinal reflection, and serve as emotionally resonant tools for navigating complex topics like HIV, consent, and stigma. The study suggests that podcasts may help complement traditional sexual health communication by offering anonymity, flexibility, and emotionally engaging storytelling.

The findings indicate that young people are not passive recipients but active respondents and critical contributors to health promotion design. Participants’ suggestions frequently reflected a desire for podcast content that is inclusive, culturally responsive, and emotionally authentic, pointing to the potential value of adopting more participatory and youth-informed approaches. As an exploratory qualitative study, these findings should be interpreted as providing contextualised insights rather than generalisable evidence of impact. While the study acknowledges methodological limitations, it supports the view that podcasting is a potentially promising, adaptable, and scalable medium for advancing equitable and youth-centred health promotion, particularly when integrated thoughtfully with local health strategies, such as those led by NSW Health. Overall, this research contributes preliminary practical and conceptual insights to evolving models of youth-centred digital health engagement.

## Supporting information

S1 Appendix Appendix_A. (PDF)

S2 Appendix Appendix_B. (PDF)

S3 Appendix Appendix_C. (PDF)

S4 Appendix Appendix_D. (PDF)

## References

[1] Morris JL, Rushwan H. Adolescent sexual and reproductive health: The global challenges. Int J Gynaecol Obstet. 2015;131 Suppl 1:S40-2. doi: 10.1016/j.ijgo.2015.02.006 26433504

[2] WHO. Defining Sexual Health. 2025. https://www.who.int/teams/sexual-and-reproductive-health-and-research/key-areas-of-work/sexual-health/defining-sexual-health

[3] Brown E, Lo Monaco S, O’Donoghue B, Nolan H, Hughes E, Graham M, et al. Improving the Sexual Health of Young People (under 25) in High-Risk Populations: A Systematic Review of Behavioural and Psychosocial Interventions. Int J Environ Res Public Health. 2021;18(17):9063. doi: 10.3390/ijerph18179063 34501652 PMC8430747

[4] Jefferson IS, Robinson SK, Tung-Hahn E, Schumann R, Marrero-Conti S, Walton JM, et al. Assessing and Improving the Knowledge of Sexually Transmitted Infections among High School Adolescents. Dermatol Res Pract. 2021;2021:6696316. doi: 10.1155/2021/6696316 33953742 PMC8057898

[5] Patterson SP, Hilton S, Flowers P, McDaid LM. What are the barriers and challenges faced by adolescents when searching for sexual health information on the internet? Implications for policy and practice from a qualitative study. Sex Transm Infect. 2019;95(6):462–7. doi: 10.1136/sextrans-2018-053710 31040251 PMC6706277

[6] McConnell EA, Janulis P, Phillips G 2nd, Truong R, Birkett M. Multiple Minority Stress and LGBT Community Resilience among Sexual Minority Men. Psychol Sex Orientat Gend Divers. 2018;5(1):1–12. doi: 10.1037/sgd0000265 29546228 PMC5846479

[7] Australian Government Department of Health and Aged Care. Fifth National Sexually Transmissible Infections Strategy 2024–2030. 2024. https://www.health.gov.au/resources/publications/fifth-national-sexually-transmissible-infections-strategy-2024-2030?language=en

[8] UNSW Kirby Institute. Sexually transmissible infections are on the rise in Australia, with syphilis rates tripling over the decade. 2023. https://www.kirby.unsw.edu.au/news/sexually-transmissible-infections-are-rise-australia-syphilis-rates-tripling-over-decade

[9] Waling A, Farrugia A, Fraser S. Embarrassment, Shame, and Reassurance: Emotion and Young People’s Access to Online Sexual Health Information. Sex Res Social Policy. 2023;20(1):45–57. doi: 10.1007/s13178-021-00668-6 35035599 PMC8743101

[10] Khatri RB, Assefa Y. Access to health services among culturally and linguistically diverse populations in the Australian universal health care system: issues and challenges. BMC Public Health. 2022;22(1):880. doi: 10.1186/s12889-022-13256-z 35505307 PMC9063872

[11] Garrett CC, Hocking J, Chen MY, Fairley CK, Kirkman M. Young people’s views on the potential use of telemedicine consultations for sexual health: results of a national survey. BMC Infect Dis. 2011;11:285. doi: 10.1186/1471-2334-11-285 22026640 PMC3213067

[12] Rohrbach LA, Berglas NF, Jerman P, Angulo-Olaiz F, Chou C-P, Constantine NA. A Rights-Based Sexuality Education Curriculum for Adolescents: 1-Year Outcomes From a Cluster-Randomized Trial. J Adolesc Health. 2015;57(4):399–406. doi: 10.1016/j.jadohealth.2015.07.004 26403840

[13] Whitehead L, Talevski J, Fatehi F, Beauchamp A. Barriers to and Facilitators of Digital Health Among Culturally and Linguistically Diverse Populations: Qualitative Systematic Review. J Med Internet Res. 2023;25:e42719. doi: 10.2196/42719 36853742 PMC10015358

[14] Centre for Population Health. NSW Sexually Transmissible Infections Strategy 2022-2026. Sydney, Australia. 2022. https://www.health.nsw.gov.au/sexualhealth/Pages/nsw-sti-strategy.aspx

[15] Robins B, Delaney T, Maher C, Singh B. Podcasts as a tool for promoting health-related behaviours: A scoping review. Digit Health. 2024;10:20552076241288630. doi: 10.1177/20552076241288630 39403714 PMC11472369

[16] Statista. Share of monthly podcast listeners in Australia in 2023, by age group. 2024. https://www.statista.com/statistics/1453181/australia-monthly-podcast-listeners-by-age/

[17] Kissell LM, Coley KC, Khieu AS, Bunk EJ, Herbert SMC, Carroll JC. Podcasts as a Method to Deliver Education on Stigma Surrounding Opioid Use Disorder. Pharmacy (Basel). 2022;10(6):161. doi: 10.3390/pharmacy10060161 36548318 PMC9781876

[18] Caddyshack. Caddyshack. 2025. https://www.caddyshackproject.com/about-caddyshack-project

[19] Keegan J, Cooper SC, Porter A, Ciervo C, Khalid R. Unlearning and relearning sexuality: a qualitative exploration of The Sex Wrap, a sex education podcast. Sex Health. 2023;20(6):531–7. doi: 10.1071/SH23109 37743095

[20] Leite PL, Torres FAF, Pereira LM, Bezerra AdM, Machado LDS, Silva MRFd. Construction and validation of podcast for teen sexual and reproductive health education. Rev Lat Am Enfermagem. 2022;30(spe):e3706. doi: 10.1590/1518-8345.6263.3706 36197393 PMC9647944

[21] Zou Y, Sharpe A, Demant D. The effectiveness of podcasts in promoting health among young people: a scoping review. Health Promot Int. 2025;40(4):daaf102. doi: 10.1093/heapro/daaf102 40607512 PMC12223501

[22] O’Brien BC, Harris IB, Beckman TJ, Reed DA, Cook DA. Standards for reporting qualitative research: a synthesis of recommendations. Acad Med. 2014;89(9):1245–51. doi: 10.1097/ACM.0000000000000388 24979285

[23] UTS. Zoom is a high quality video and audio online conferencing tool for desktops, tablets and smartphones. Accessed 2025 May 5. https://www.uts.edu.au/for-students/current-students/managing-your-course/using-uts-systems/software-available-students/zoom

[24] AARNet. AARNet’s response to Zoom security and privacy issues. 2020. https://aarnet.edu.au/aarnets-response-to-zoom-security-and-privacy-issues

[25] AARNet. Zooming beyond COVID: Did the pace of learning really Zoom, or just the tools? 2023. https://aarnet.edu.au/zooming-beyond-covid-did-the-pace-of-learning-really-zoom-or-just-the-tools

[26] Braun V, Clarke V. Using thematic analysis in psychology. Qualitative Research in Psychology. 2006;3(2):77–101. doi: 10.1191/1478088706qp063oa

[27] Jowsey T, Deng C, Weller J. General-purpose thematic analysis: a useful qualitative method for anaesthesia research. BJA Educ. 2021;21(12):472–8. doi: 10.1016/j.bjae.2021.07.006 34840819 PMC8606608

[28] Towner E, Chierchia G, Blakemore S-J. Sensitivity and specificity in affective and social learning in adolescence. Trends Cogn Sci. 2023;27(7):642–55. doi: 10.1016/j.tics.2023.04.002 37198089

[29] Papacharissi Z. Affective publics and structures of storytelling: sentiment, events and mediality. Information, Communication & Society. 2016;19(3):307–24. doi: 10.1080/1369118x.2015.1109697

[30] Bandura A. Self-efficacy: The exercise of control. W H Freeman/Times Books/ Henry Holt & Co.; 1997.

[31] Mitchell KR, Lewis R, O’Sullivan LF, Fortenberry JD. What is sexual wellbeing and why does it matter for public health? Lancet Public Health. 2021;6(8):e608–13. doi: 10.1016/S2468-2667(21)00099-2 34166629 PMC7616985

[32] Porter AW, Cooper SC, Palmedo PC, Wojtowicz N, Chong J, Maddalon M. Podcasts and Their Potential to Improve Sexual Health Literacy in Adolescents and Young Adults. American Journal of Sexuality Education. 2022;17(1):125–36. doi: 10.1080/15546128.2021.1987365

[33] Alyafei A, Easton-Carr R. The Health Belief Model of Behavior Change. StatPearls Publishing LLC; 2024. 39163427

[34] Alimoradi S, Patrick RNC, Dickerson DE. Rethinking Focus Groups: Inherent Issues and How to Measure Them. Proceedings of the Human Factors and Ergonomics Society Annual Meeting. 2025;69(1):124–9. doi: 10.1177/10711813251361005

[35] Adeyeba M, Calvetti S, Lockett G, Sostre J, Slay L, Goldbach JT, et al. Intersecting Identities: Exploring stigma, minority stress, resilience, and identity in sexual and gender diverse youths of color. SSM Ment Health. 2025;7:100458. doi: 10.1016/j.ssmmh.2025.100458 40630798 PMC12237429

[36] Goundar PR. Researcher Positionality: Ways to Include it in a Qualitative Research Design. International Journal of Qualitative Methods. 2025;24. doi: 10.1177/16094069251321251

[37] Mansouri F, Beaman L, Hussain S. Multicultural Realities: The Everyday Experiences of Migrant Youth in Melbourne, Toronto and Birmingham. Journal of Intercultural Studies. 2025;46(4):629–44. doi: 10.1080/07256868.2025.2565758

