# Supplementary material for: Exploring the effectiveness of podcasts in improving sexual health among young people: Findings from a qualitative study
Source: PLoS One. 2026 Mar 27;21(3):e0343514. doi: 10.1371/journal.pone.0343514 (PMC13029784; doi:10.1371/journal.pone.0343514)
Supplement: S2 Appendix — (PDF) [file pone.0343514.s002.pdf]

Draft Focus Group Questions

Young People

1. **Engagement and Appeal:**
    - What were your first impressions of the podcasts? What did you like or dislike about them?
2. **Relevance to Your Life:**
    - How well do you think the topics discussed in the podcasts relate to your own experiences and the issues faced by young people today?
3. **Impact on Knowledge:**
    - Did listening to the podcasts change your understanding of sexual and reproductive health? If so, how?
4. **Influence on Health Behaviours:**
    - After listening to the podcasts, did you feel more motivated to take any actions regarding your own sexual or reproductive health?
5. **Accessibility and Usability:**
    - How easy was it for you to access the podcasts, and do you think podcasts are a good way for young people to learn about health topics?
6. **Preference for Podcast Format:**
    - What do you think about the length and style of the podcasts? Do you prefer short episodes or more detailed, longer discussions?
7. **Improvements:**
    - What changes or improvements would you suggest to make the podcasts more engaging or informative for young people?
8. **Health Promotion through Podcasts:**
    - In your opinion, are podcasts an effective way to promote health messages to young people? Why or why not?
9. **Sexual and Reproductive Health Focus:**
    - How well do you think the podcasts addressed sexual and reproductive health topics? Were there any areas you feel were missing or underexplored?
10. **Broader Use of Podcasts:**

- Can you think of other health topics that could benefit from being covered in a podcast format? What would make these podcasts successful?
