# Supplementary material for: Exploring the effectiveness of podcasts in improving sexual health among young people: Findings from a qualitative study
Source: PLoS One. 2026 Mar 27;21(3):e0343514. doi: 10.1371/journal.pone.0343514 (PMC13029784; doi:10.1371/journal.pone.0343514)
Supplement: S3 Appendix — (PDF) [file pone.0343514.s003.pdf]

UTS UNIVERSITY OF TECHNOLOGY SYDNEY

PARTICIPANT INFORMATION SHEET

Evaluation of the Caddyshack Program in the Illawarra Shoalhaven Local Health District

WHO IS CONDUCTING THIS RESEARCH?

This research is conducted by Mr Yixuan Zou, a Bachelor of Public Health Honours student at UTS, supervised by Dr Daniel Demant and Associate Professor Nikki Percival from the School of Public Health at the University of Technology Sydney.

WHAT IS THE RESEARCH ABOUT?

This research is about evaluating the On the Couch Podcast developed and distributed by the Caddyshack Program in the Illawarra Shoalhaven Local Health District. The Caddyshack Program is a sexual health program that promotes a positive approach to sex and sexuality for young people (aged 16 - 29) and the broader community.

WHY HAVE I BEEN INVITED?

You are invited to participate in this research project. You have been invited because you demonstrated interest in participating in this research project and are part of the groups targeted by the podcasts that are being evaluated: young people and those who use the podcasts as part of their professional role or development in areas with relevance to sexual and reproductive health.

FUNDING

Funding for this project has been received from the Illawarra Shoalhaven LHD. This research project has been designed to make sure the researchers interpret the results in a fair and appropriate way, independently from the Illawarra Shoalhaven LHD.

WHAT DOES MY PARTICIPATION INVOLVE?

If you decide to participate, we will invite you to participate in a focus group with Mr Yixuan Zou and one of his supervisors. The focus group will take place at the UTS Campus. The focus group will last approximately 45 to 60 minutes. During the focus group, we will discuss the On the Couch podcasts and ask about your opinion on these, including how these may be improved. You will be asked to listen to one to two of the podcasts prior to attending the focus group. You will receive a retail voucher of $30 for your participation.

Yes, there are some risks/inconvenience. These may include the time you need to spend to participate in this research. We don’t expect this interview to cause any other harm or discomfort. However, if you experience discomfort or distress during the focus group, please let us know, and we take a break or you can leave the focus group altogether. If you experience distress or discomfort after the conclusion of the interview, please contact your family doctor or physician. You can also contact the student counselling services of your university or TAFE if you are currently enrolled at a university or TAFE. If these services are not available to you, you can contact Beyond Blue on 130022466 or through their website beyondblue.org.au.

It is important to be aware that while we take confidentiality seriously, there is a potential risk of breaches of confidentiality in any group setting like this focus group. Therefore, we request all participants to respect the privacy of others and keep the information shared within the group confidential, as some of it may be personal and sensitive.

While we intend this study to further knowledge and recommend improvements to the Caddyshack Program in the future, there will be no clear benefit to you from your participation in this research.

DO I HAVE TO TAKE PART IN THIS RESEARCH PROJECT?

Participation in this study is voluntary. It is completely up to you whether or not you decide to take part. If you decide not to participate, or to withdraw from the study, it will not affect your relationship with the researchers or the University of Technology Sydney or the Illawarra Shoalhaven LHD or the University of Technology Sydney.

WHAT IF I WITHDRAW FROM THIS RESEARCH PROJECT?

If you wish to withdraw from the study once it has started, you can do so at any time without having to give a reason by indicating this to the researcher. However, it may not be possible to withdraw the data you have provided until this point in the focus group from the study results due to the interactive nature of a focus group.

WHAT WILL HAPPEN TO INFORMATION ABOUT ME?

By providing consent to the research team collecting and using personal information about you for the research project. All this information will be treated confidentially. The focus group will be transcribed and the recording file will be deleted afterwards. Any identifying information (e.g., names) in the transcripts will be removed. All files associated with this research will be stored on secured, password-protected hard drives at the University of Technology Sydney and will not be shared with the Illawarra Shoalhaven LHD. Your information will only be used for the purpose of this research project, and it will only be disclosed with your permission, except as required by law.

It is anticipated that the results of this research project will be published and/or presented in a variety of forums. In any publication and/or presentation, information will be provided in such a way that you cannot be identified, except with your permission. These publications (e.g., peer-reviewed manuscripts, conference presentations or pre-prints) will be accessible to you through the UTS OPUS portal once available.

In accordance with relevant Australian and/or NSW Privacy laws, you have the right to request access to the information about you that is collected and stored by the research team. You also have the right to request that any information with which you disagree be corrected. Please inform the research team member named at the end of this document if you would like to access your information. Please do note that this may not be possible if identifying information has been removed.

WHAT IF I HAVE ANY QUERIES OR CONCERNS?

If you have any queries or concerns about the research that you think we can help you with, please feel free to contact us:

- Dr Daniel Demant, E: daniel.demant@uts.edu.au; T: 02 9514 5499

- Associate Professor Nikki Percival, E: nikki.percival@uts.edu.au; T: 02 9514 5232

This study has been approved by the Greater Western Human Research Ethics Committee. If you have any concerns or complaints about the conduct of the research study, you may contact the Executive Officer of the Ethics Committee on 02 6330 5948 and quote: 2023/ETH02655

CONSENT FORM

Evaluation of the Caddyshack Program in the Illawarra Shoalhaven Local Health District

I ____________________ *[participant's name]* agree to participate in the research project being conducted by *[give name, UTS address and contact telephone.]*. I understand that funding for this research has been provided by the Illawarra Shoalhaven Local Health District.

I have read the Participant Information Sheet or someone has read it to me in language that I understand.

I understand the purposes, procedures and risks of the research as described in the Participant Information Sheet.

I have had an opportunity to ask questions and I am satisfied with the answers I have received.

I freely agree to participate in this research project as described and understand that I am free to withdraw at any time without affecting my relationship with the researchers or the University of Technology Sydney or any other organisation

I understand that I will be given a signed copy of this document to keep.

I understand that the focus group will be recorded and transcribed.

I am aware that I can contact *[researcher's name]* or the Executive Officer of the Greater Western Uman Research Ethics Committee if I have any concerns about the research.

____ ____/____/____
Name and Signature [participant] Date

____ ____/____/____
Name and Signature [researcher or delegate] Date
