# Supplementary material for: Exploring the effectiveness of podcasts in improving sexual health among young people: Findings from a qualitative study
Source: PLoS One. 2026 Mar 27;21(3):e0343514. doi: 10.1371/journal.pone.0343514 (PMC13029784; doi:10.1371/journal.pone.0343514)
Supplement: S4 Appendix — (PDF) [file pone.0343514.s004.pdf]

UTS UNIVERSITY OF TECHNOLOGY SYDNEY

NSW GOVERNMENT Health Illawarra Shoalhaven Local Health District

Join the Conversation on Sexual Health Education!

- University of Technology (UTS) is conducting a study of the "On The Couch" podcast, part of the Caddyshack Project Program by Illawarra Shoalhaven Local Health District. This research, led by UTS Honours Student, Yixuan Zou, aims to assess how the podcast resonates with young people and the broader community.

- On The Couch hosts guests to discuss and explore current social themes and sex positive topics where they collaborate with experts, practitioners, authors, advocates, and influencers to share stories and insights that matter.

- We are looking for young people (18 to 24) or professionals working in a field relevant to sexual and reproductive health to participate in focus groups held on remote Zoom. By understanding its impact and gathering feedback, we can enhance educational tools that promote positive sexual health practices. This is a great opportunity to explore how digital media can support effective health education. Each participant will receive $30 value of voucher.

- *Please scan the QR code directly to get involved. We really appreciate your participation, if you have any questions please contact Email : Yixuan.Zou@student.uts.edu.au*
